# Supplementary figures and images for: Evaluating the impacts of microplastics on agricultural soil physical, chemical properties, and toxic metal availability: An emerging concern for sustainable agriculture
Source: PLoS One. 2025 Feb 6;20(2):e0304811. doi: 10.1371/journal.pone.0304811 (PMC11801572; doi:10.1371/journal.pone.0304811)

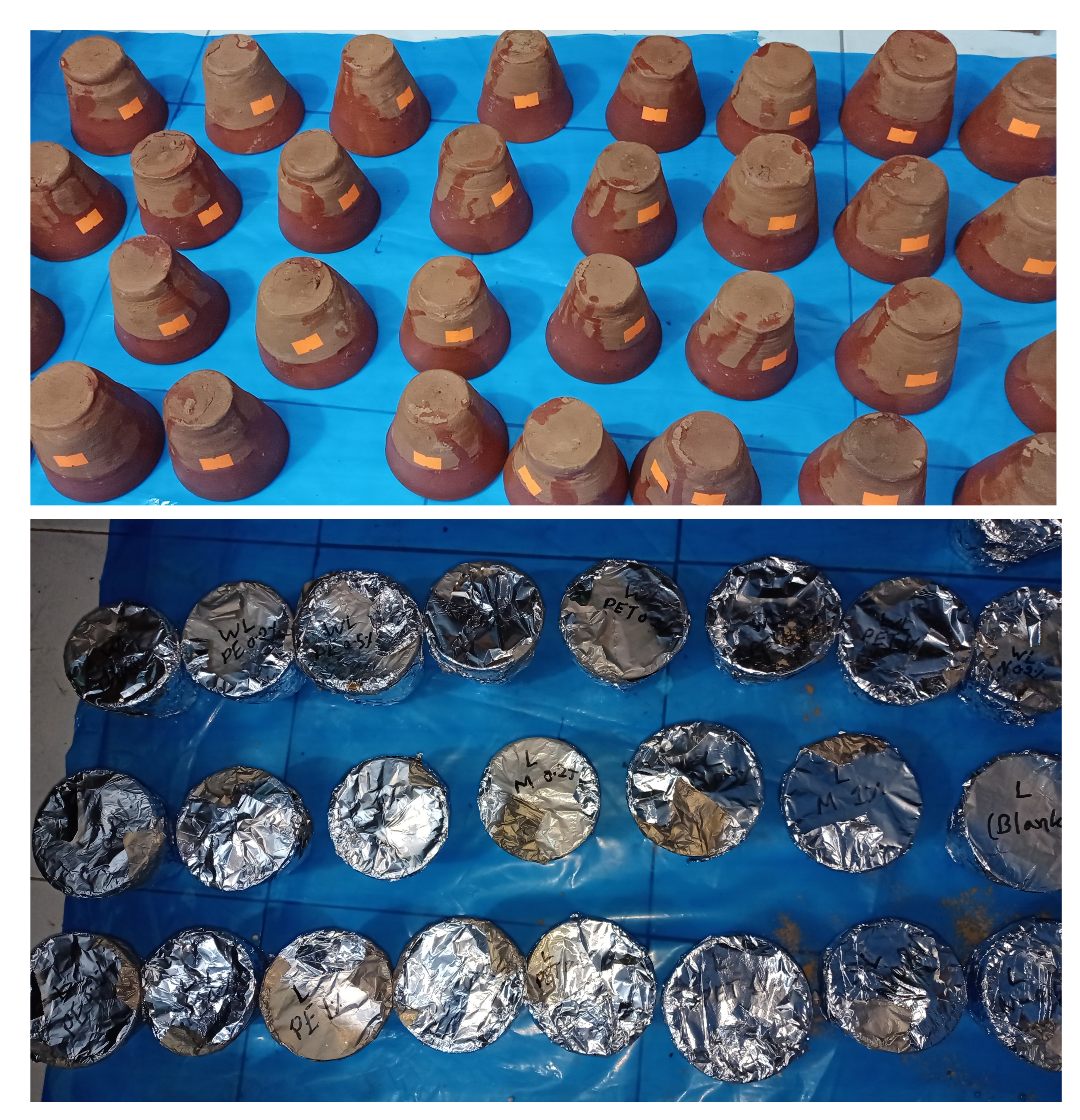

Supplement: S1 Fig — (TIF) [file pone.0304811.s001.tif]

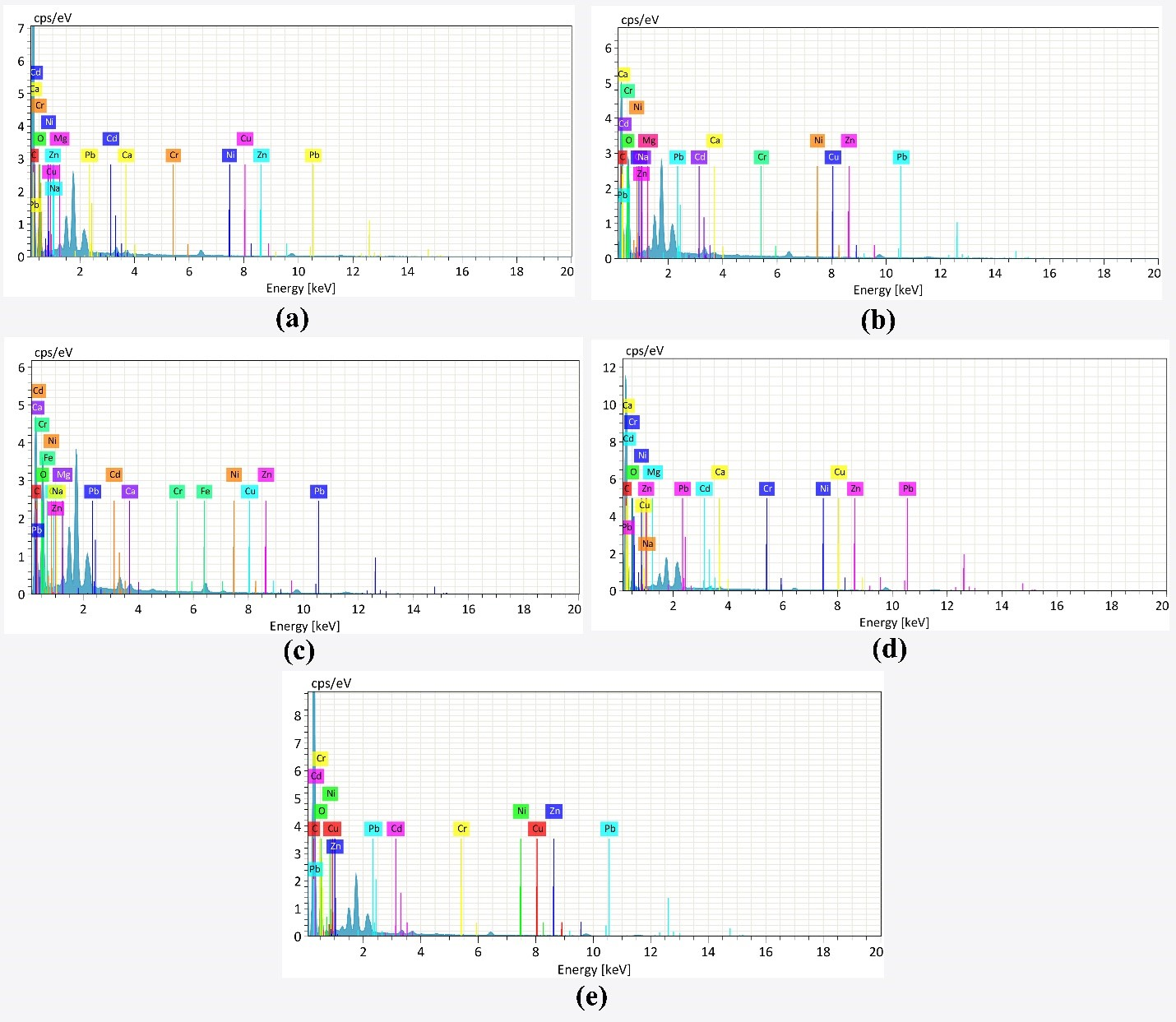

Supplement: S2 Fig — EDS of MPs after experiments (a) PET, (b), PS, (c), PE, (d) PA, (e) Mixed MPs. (TIF) [file pone.0304811.s002.tif]

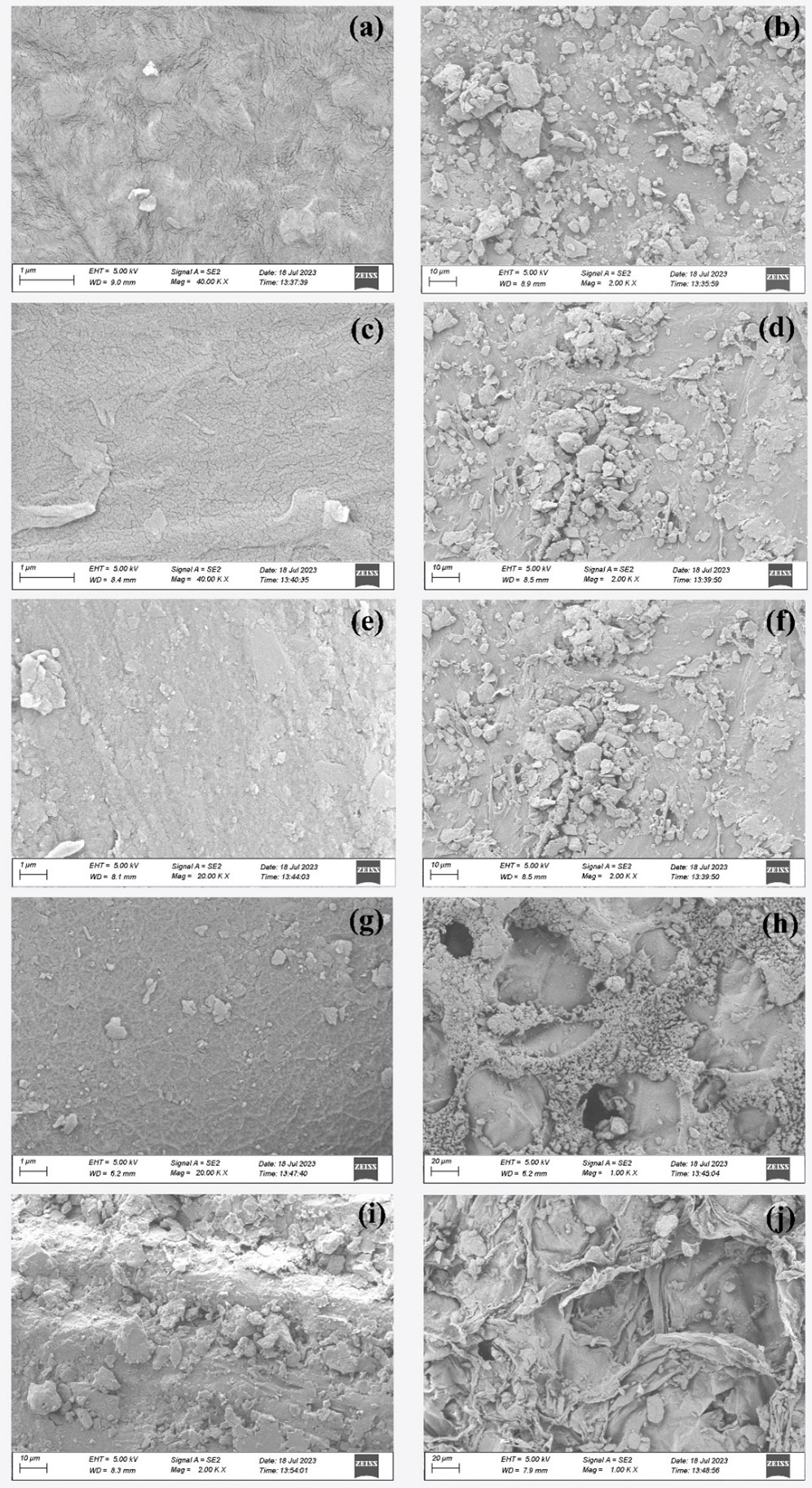

Supplement: S3 Fig — SEM of MPs after experiments for before (b) PET, (d), PS, (f), PE, (h) PA, (j) Mixed MPs and after (a) PET, (c), PS, (e), PE, (g) PA, (i) Mixed MPs. (TIF) [file pone.0304811.s003.tif]
